# First person – Diana Wall

**DOI:** 10.1242/dmm.053007

**Published:** 2026-06-02

## Abstract

First Person is a series of interviews with the first authors of a selection of papers published in Disease Models & Mechanisms, helping researchers promote themselves alongside their papers. Diana Wall is first author on ‘ *Caenorhabditis elegans* models of alternating hemiplegia of childhood have dominant neuromuscular junction defect*s*’, published in DMM. Diana is a PhD candidate in the lab of Anne Hart at Brown University, Providence, RI, USA, investigating how genetic mutations cause disease on a cellular and molecular level.

**Figure:**
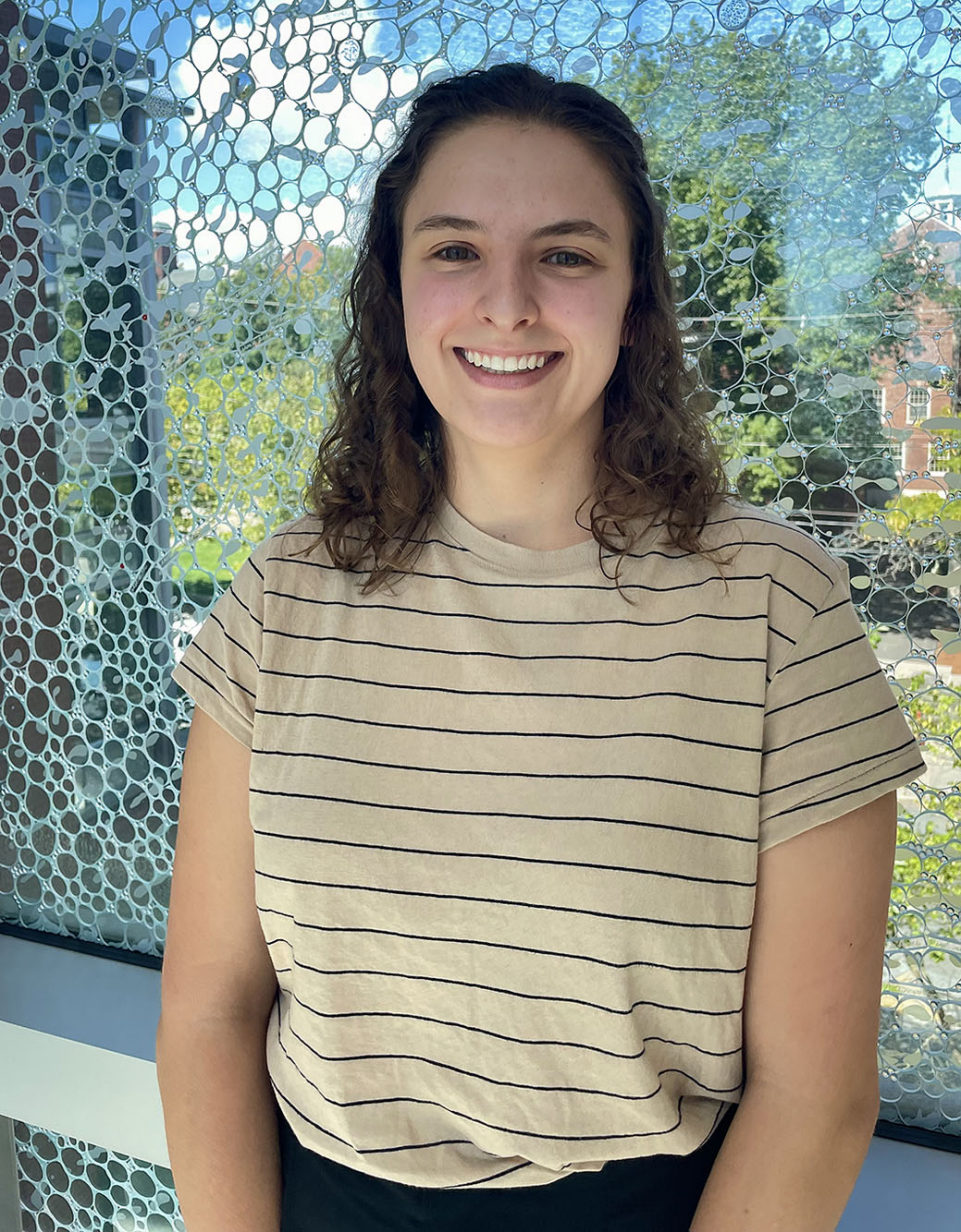
Diana Wall

**Who or what inspired you to become a scientist?**

Science was always my favorite subject in school, but I didn't realize it could be a career until I joined a lab as an undergraduate in college. I became deeply interested in research, and my advisor, Dr Kenneth Mills, encouraged me to apply to graduate school. As I enter the final year of my PhD, I am incredibly glad I chose to pursue research and become a scientist!

**What is the main question or challenge in disease biology you are addressing in this paper? How did you go about investigating your question or challenge?**

It is well established that mutations in ATP1A3, a Na^+^/K^+^ ATPase, cause a variety of rare diseases, including alternating hemiplegia of childhood (AHC). However, we do not know how these mutations disrupt cellular function and lead to disease. In our current paper, we generated and characterized *C. elegans* models of AHC for use in investigating the precise cellular and molecular mechanisms of disease.

**How would you explain the main findings of your paper to non-scientific family and friends?**

The main finding of our paper is that, in *C. elegans* models, AHC patient mutations disrupt proper communication between neurons and muscles. This disruption occurs when animals have one mutated copy and one normal copy of the gene. However, this neuromuscular impairment is not simply due to the mutated copy passively losing its function. Instead, the mutated copy is likely to actively interfere in some way, causing disruption.

*C. elegans* models of AHC are a great system to further study disease mechanisms and identify targets for novel therapeutic approaches

**What are the potential implications of these results for disease biology and the possible impact on patients?**

Currently, there are no effective therapies for AHC and related diseases beyond symptomatic management. A more precise understanding of how patient mutations disrupt cellular function will provide the fundamental basis for development of more targeted and more effective treatments. The *C. elegans* models of AHC are a great system to further study disease mechanisms and identify targets for novel therapeutic approaches.

Publishing in DMM will make our findings accessible not only to researchers studying AHC, but also to those working on other rare diseases and to the wider *C. elegans* community

**Figure:**
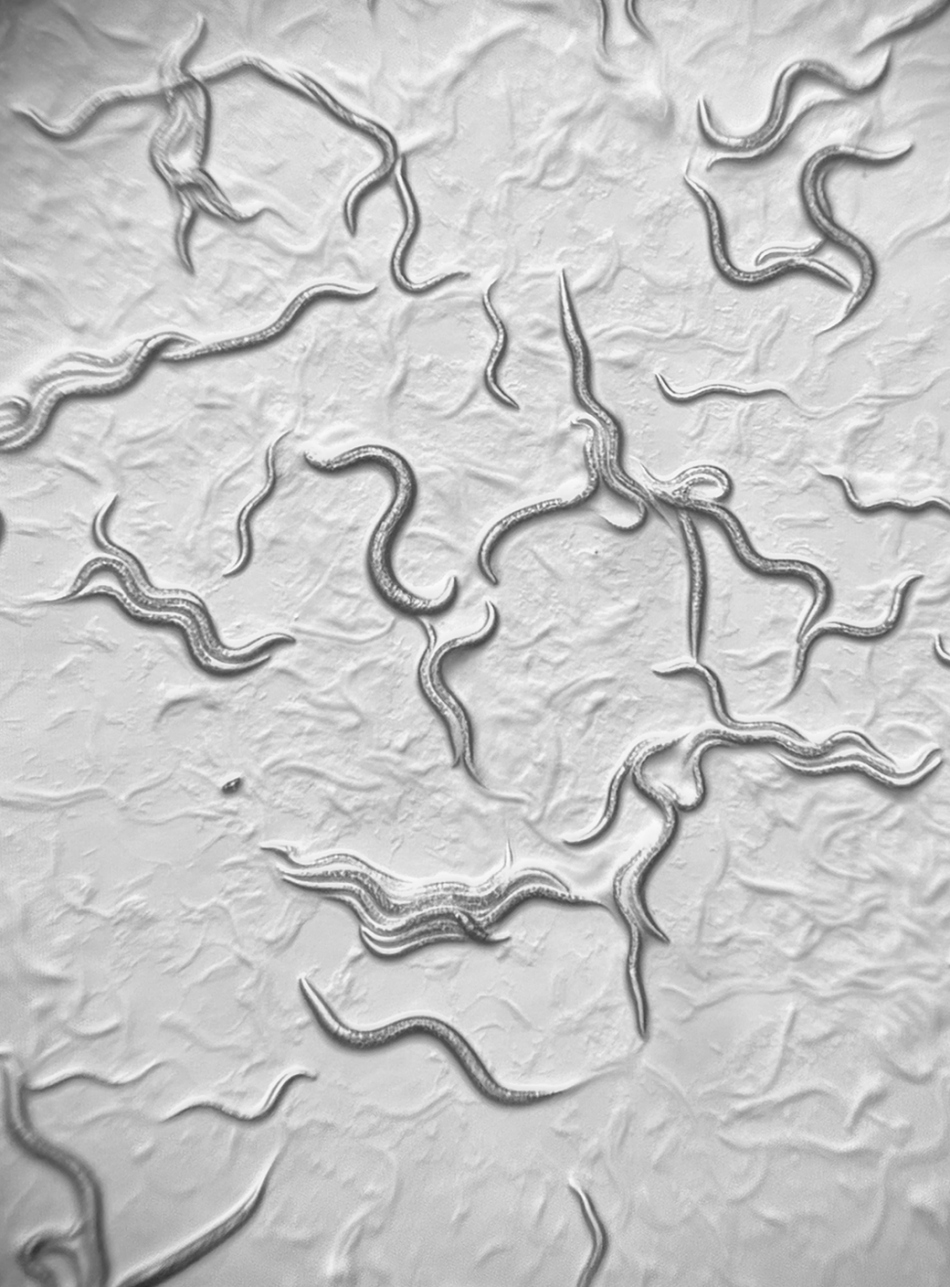
***C. elegans* models of alternating hemiplegia of childhood viewed through a dissection microscope**.

**Why did you choose DMM for your paper?**

Disease Models & Mechanisms (DMM) was the perfect fit to publish our novel *C. elegans* models of AHC. The journal upholds a rigorous peer-review process and a strong commitment to open-access, ensuring broad visibility of our work. We believe that publishing in DMM will make our findings accessible not only to researchers studying AHC, but also to those working on other rare diseases and to the wider *C. elegans* community.

**Given your current role, what challenges do you face and what changes could improve the professional lives of other scientists in this role?**

One of the biggest challenges I face personally is balancing hands-on experimental work and writing. I find myself eager to continue experiments and generate more data, which could easily take up my entire workday. However, my advisor has emphasized that data analysis and writing deserve just as much dedicated time as data collection itself. To ensure I make consistent progress on manuscripts, I have found that blocking off specific time in my calendar for writing or figure generation helps me stay focused on these tasks so that they do not pile up.

**What's next for you?**

As I wrap up my dissertation, I am excited to complete testing a candidate list of genetic modifiers in our AHC models. I am also beginning to explore potential postdoctoral positions and am interested in continuing to use *C. elegans* as a model system to study the mechanisms of disease or, more broadly, fundamental questions in biology.

**Tell us something interesting about yourself that wouldn't be on your CV**

I love doing anything crafty! Most recently, I've learned how to crochet, and my favorite things to make are sweaters.

## References

[DMM053007C1] Wall, D. A., Friedberg, A. M., Lins, J., Khalifa, R., Partipillo, S. and Hart, A. C. (2026). *Caenorhabditis elegans* models of alternating hemiplegia of childhood have dominant neuromuscular junction defects. *Dis. Model. Mech.* 19, dmm052809. 10.1242/dmm.052809 42063340 PMC13267776

